# Supplementary material for: UBQLN Family Members Regulate MYC in Lung Adenocarcinoma Cells
Source: Cancers (Basel). 2023 Jun 28;15(13):3389. doi: 10.3390/cancers15133389 (PMC10340487; doi:10.3390/cancers15133389)

Figure S1

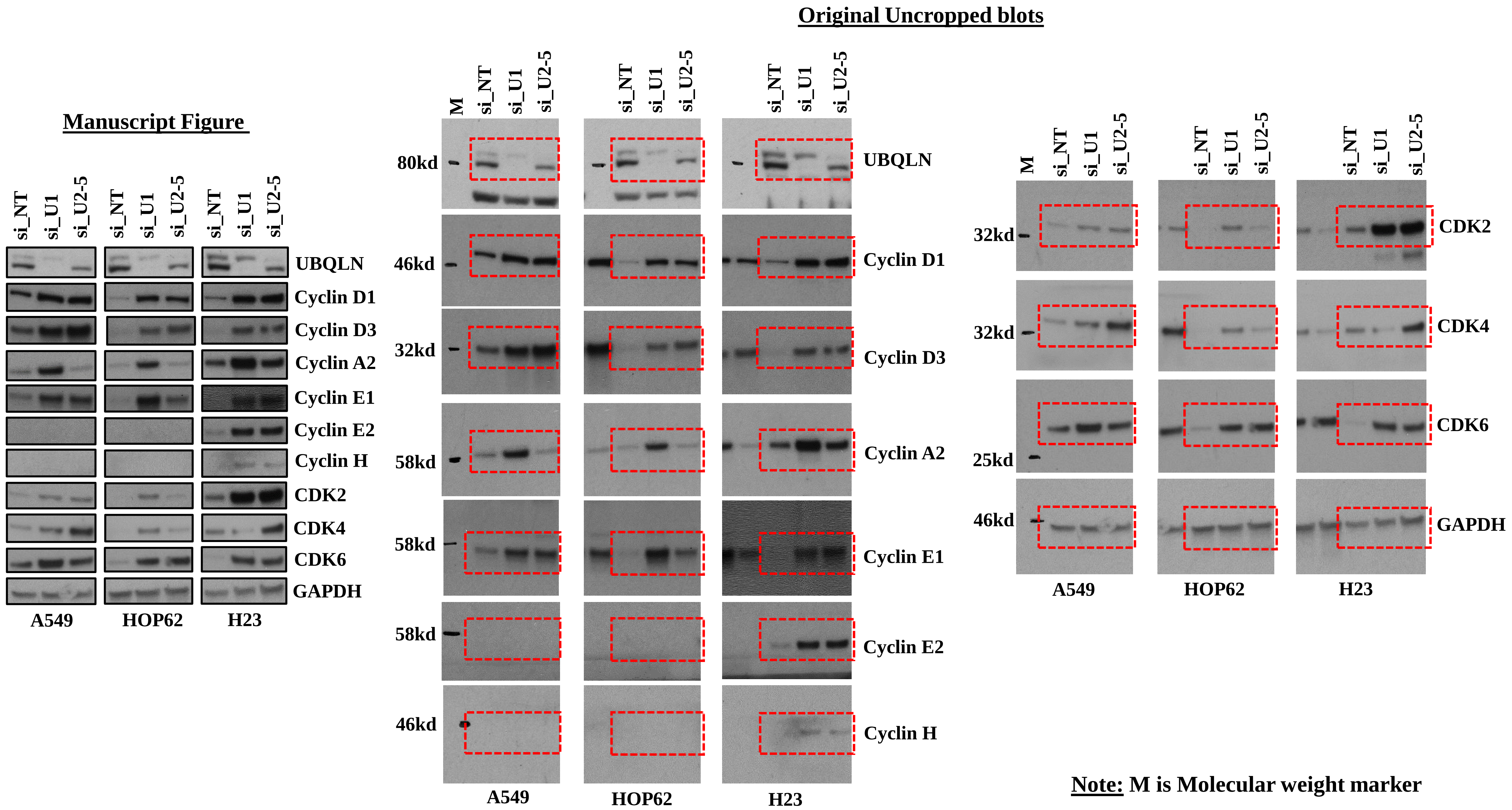

Original Uncropped blots
Manuscript Figure
si_NT
si_U1
si_U2-5
UBQLN
Cyclin D1
Cyclin D3
Cyclin A2
Cyclin E1
Cyclin E2
Cyclin H
CDK2
CDK4
CDK6
GAPDH
A549
HOP62
H23
M
80kd
46kd
32kd
58kd
58kd
58kd
46kd
32kd
32kd
25kd
46kd
Note: M is Molecular weight marker

Figure S2

Manuscript Figure

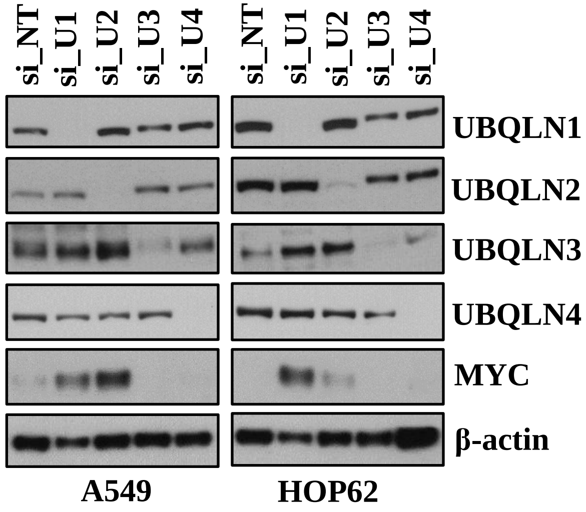

Original Uncropped blots

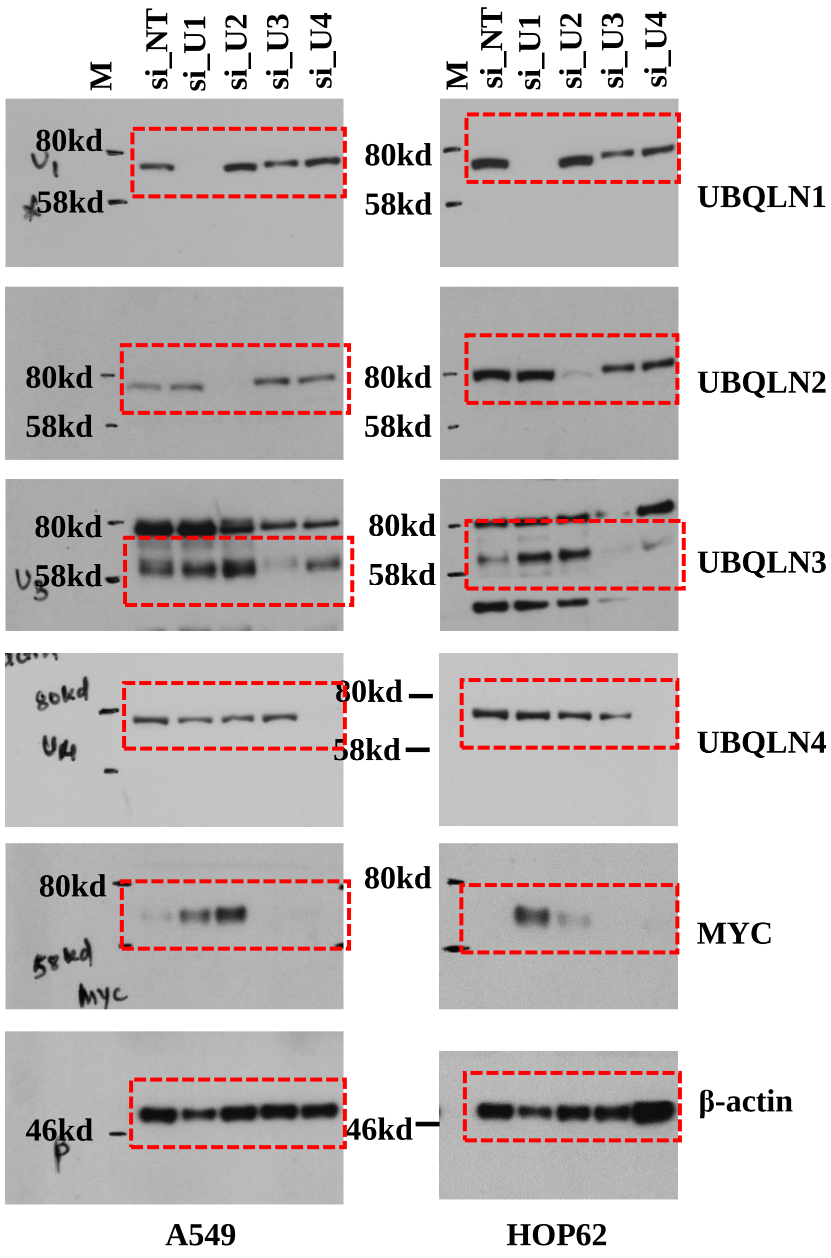

Note: M is Molecular weight marker

Figure S3A

<u>**Manuscript Figure**</u>

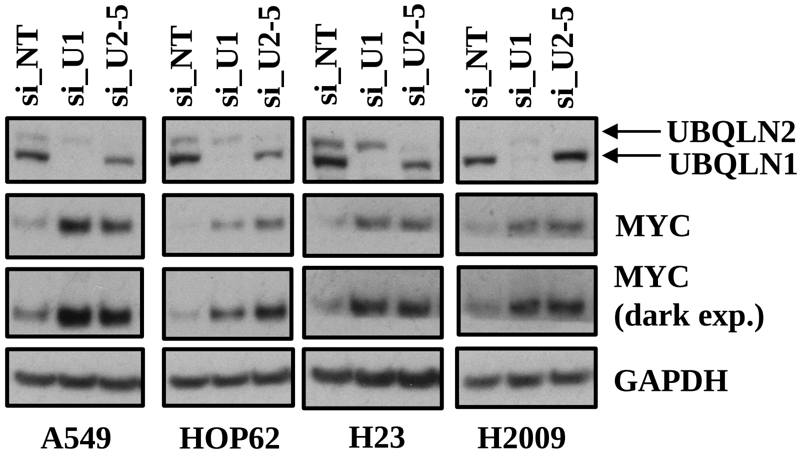

<u>**Original Uncropped blots**</u>

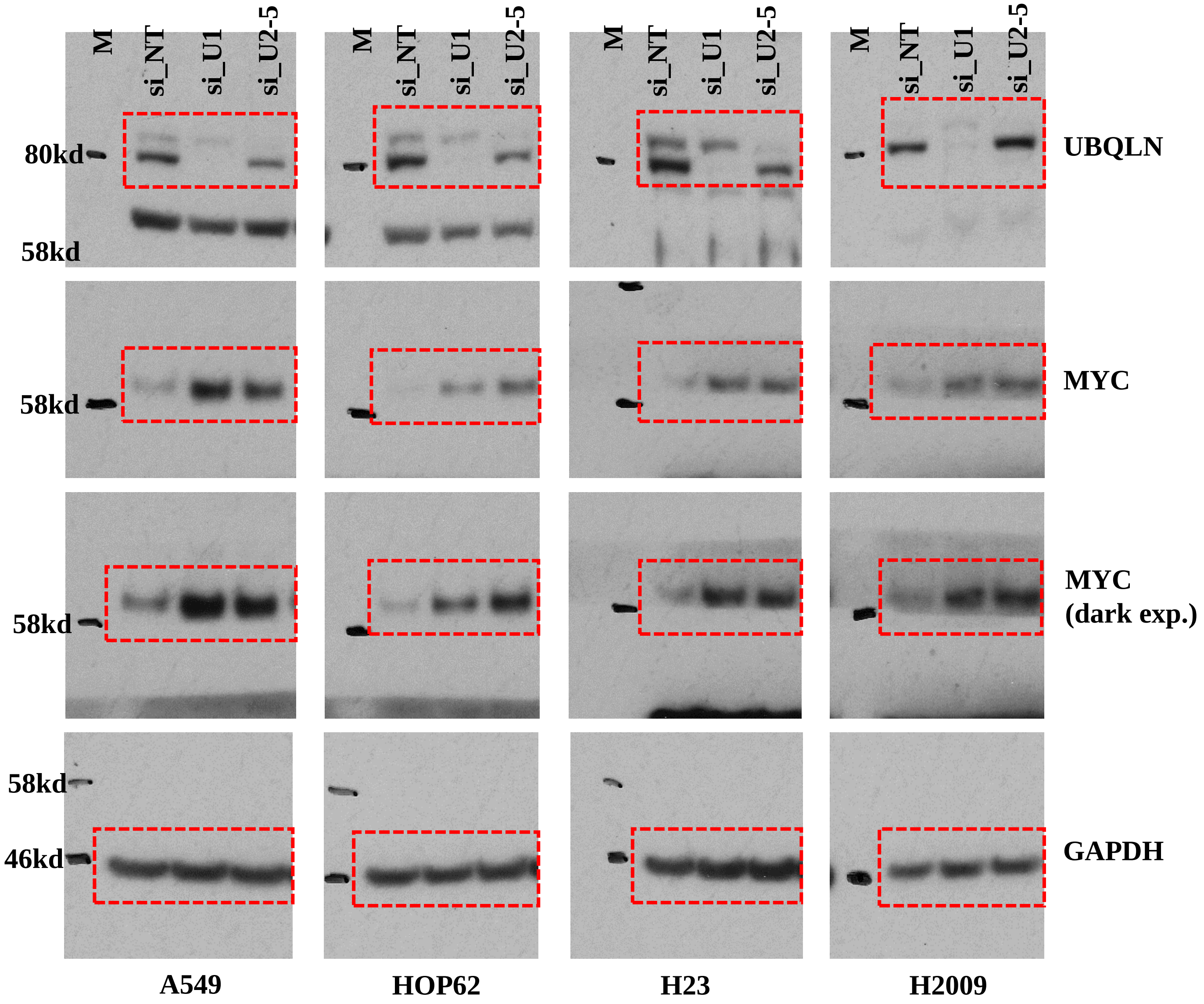

<u>**Note:**</u> **M is Molecular weight marker**

Figure S3B

Manuscript Figure

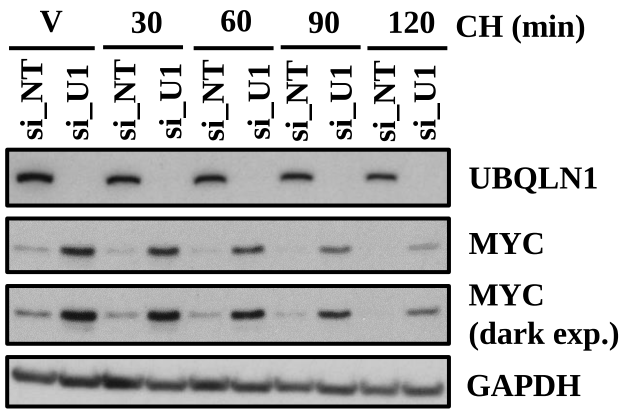

A549

Original Uncropped blots

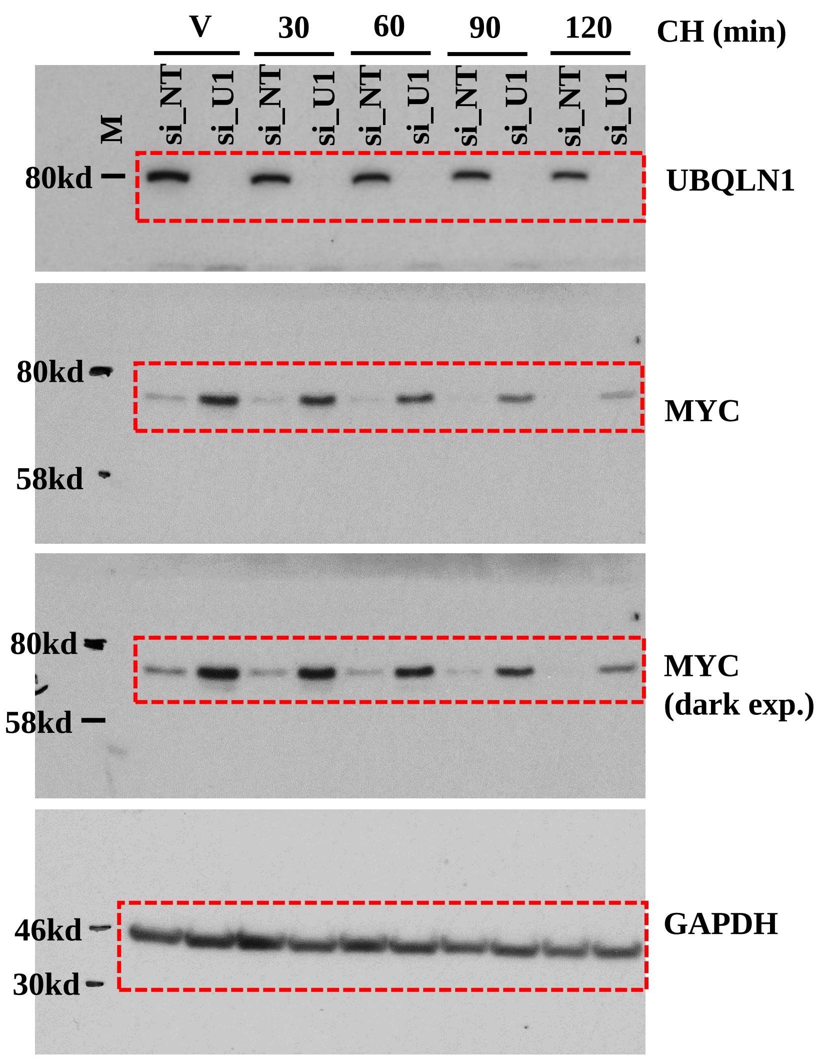

A549

Note: M is Molecular weight marker

Figure S3C

Manuscript Figure

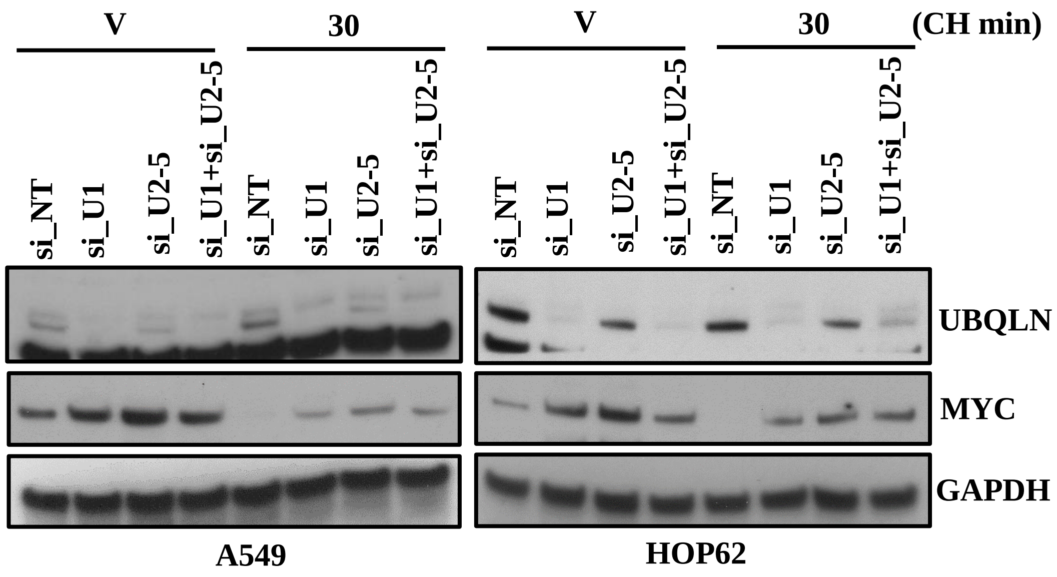

Original Uncropped blots

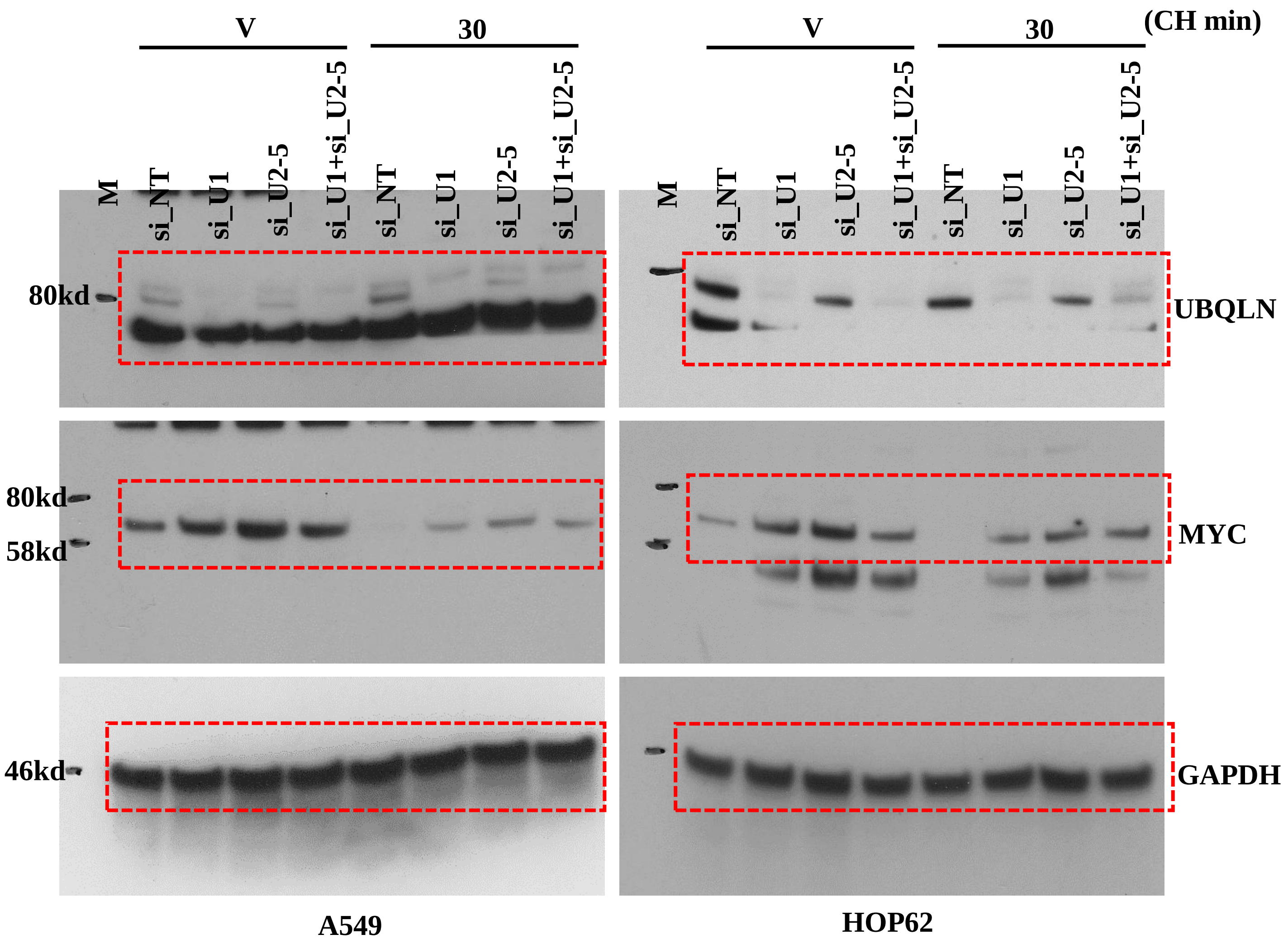

Note: M is Molecular weight marker

Figure S3D

Original Uncropped blots

Manuscript Figure

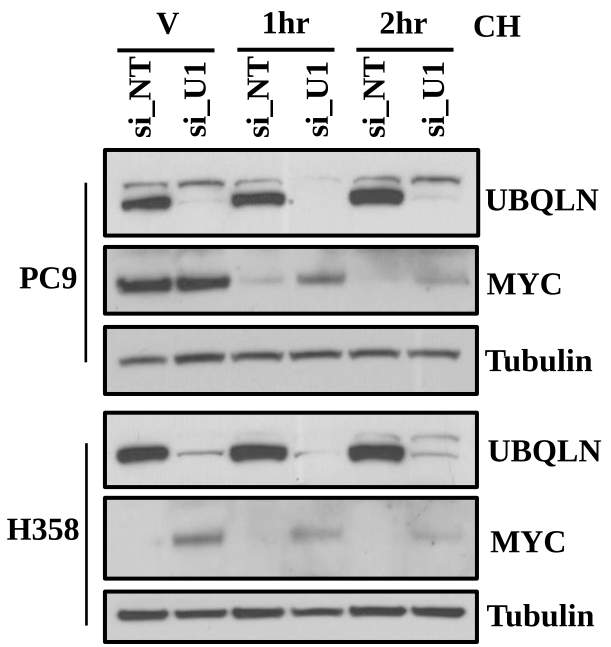

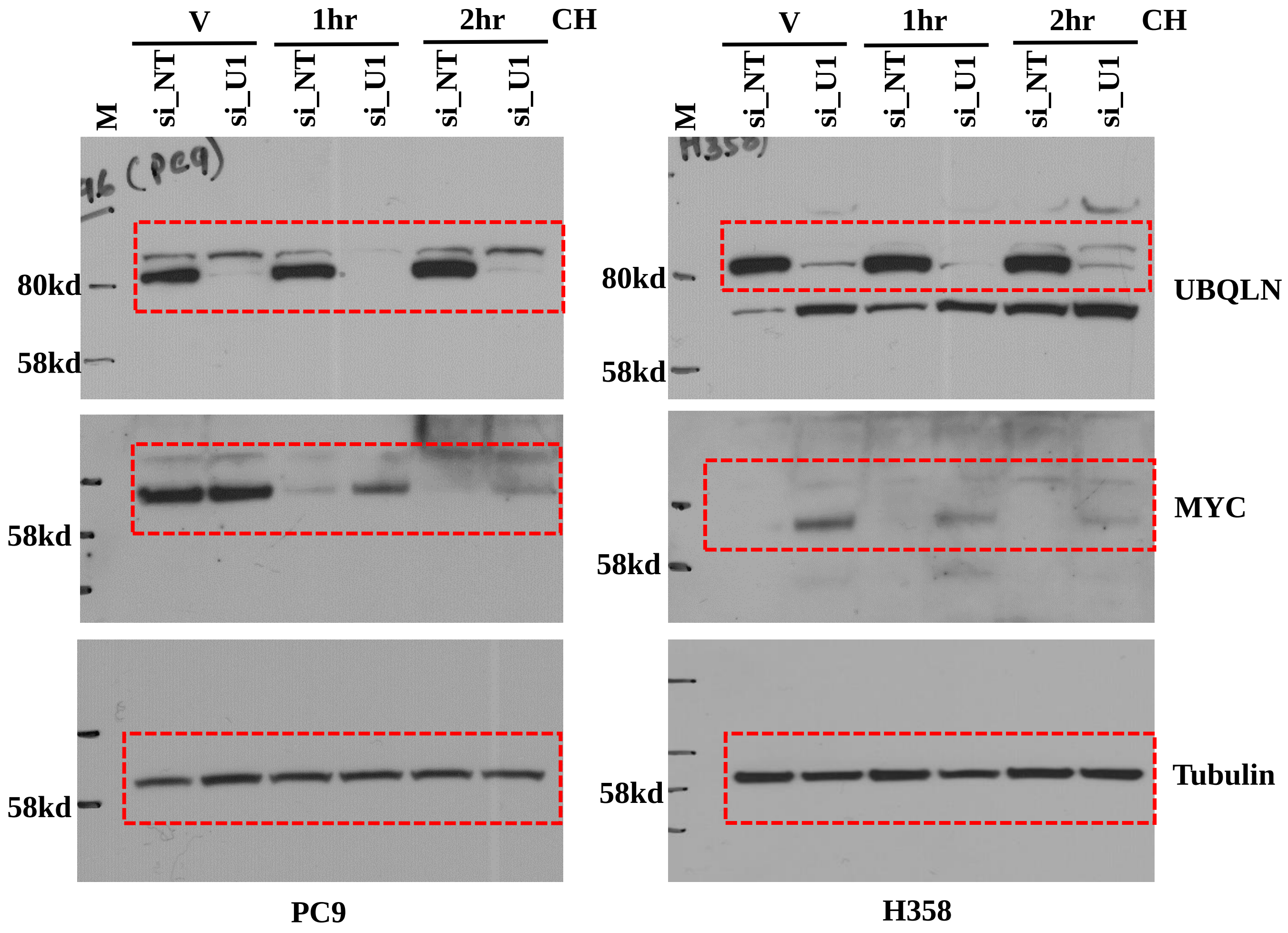

Note: M is Molecular weight marker

Figure S4A

Original Uncropped blots

Manuscript Figure

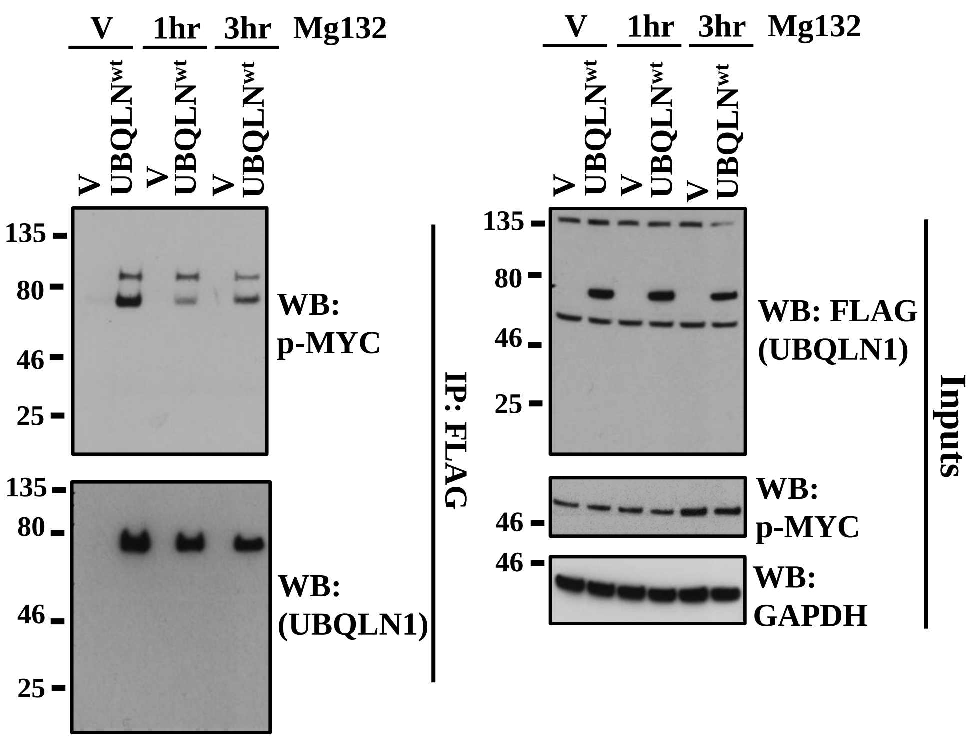

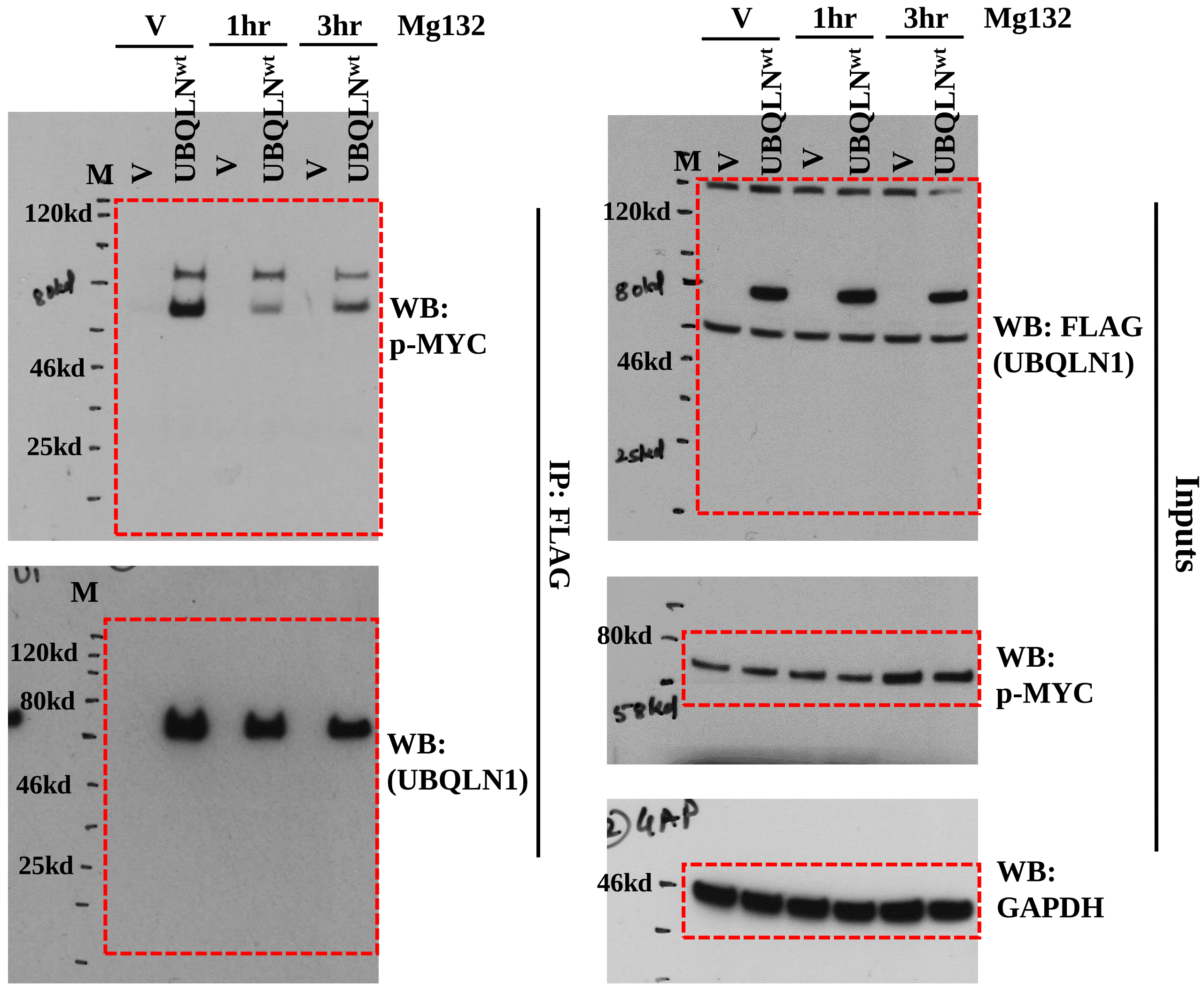

Note: M is Molecular weight marker

Figure S4B

Manuscript Figure

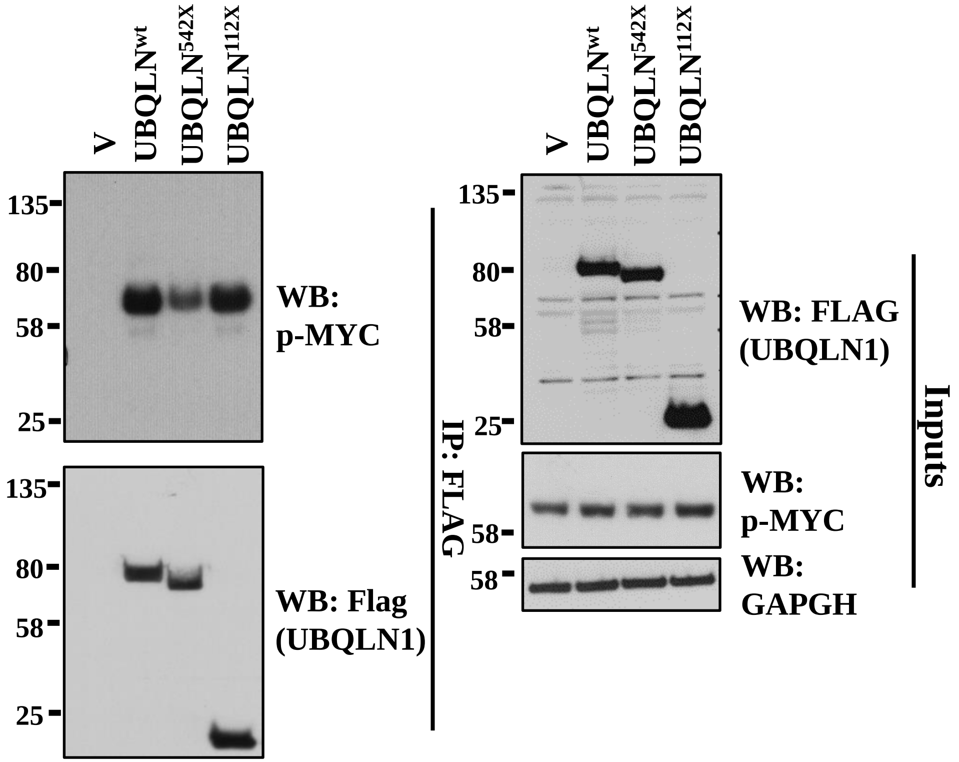

Original Uncropped blots

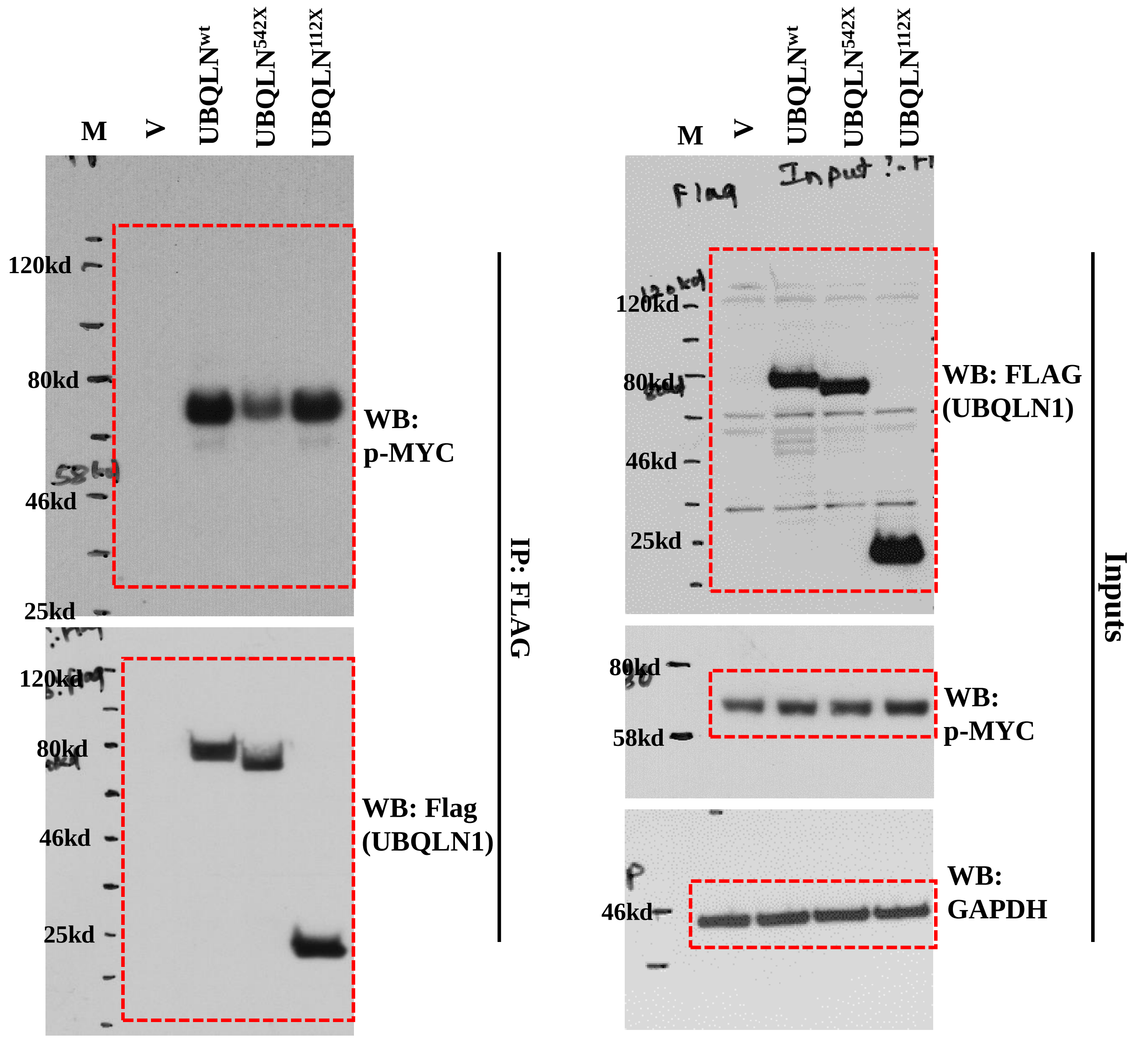

Note: M is Molecular weight marker

Figure S4C

Manuscript Figure

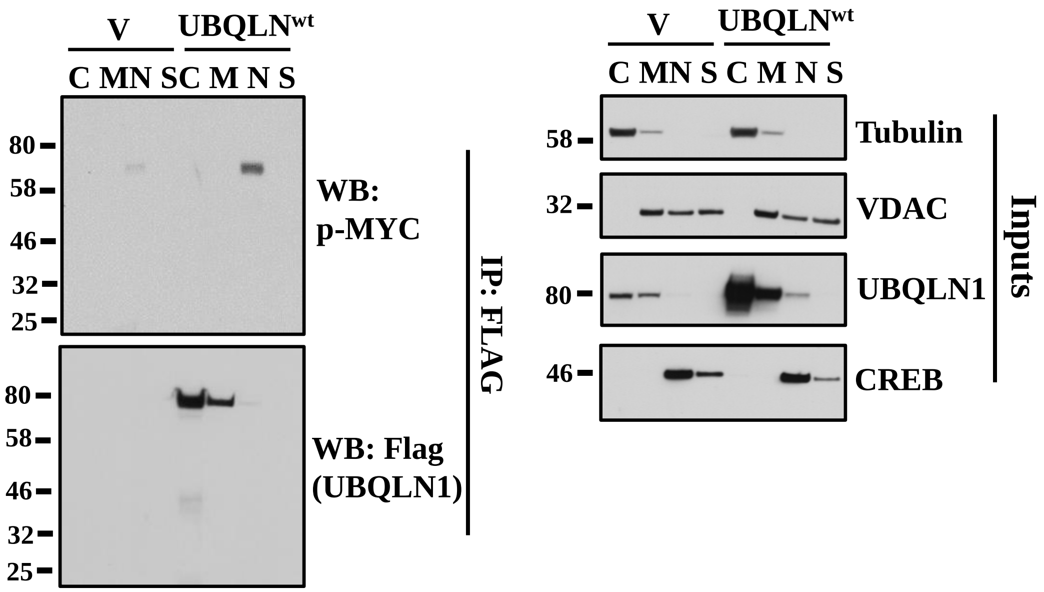

Original Uncropped blots

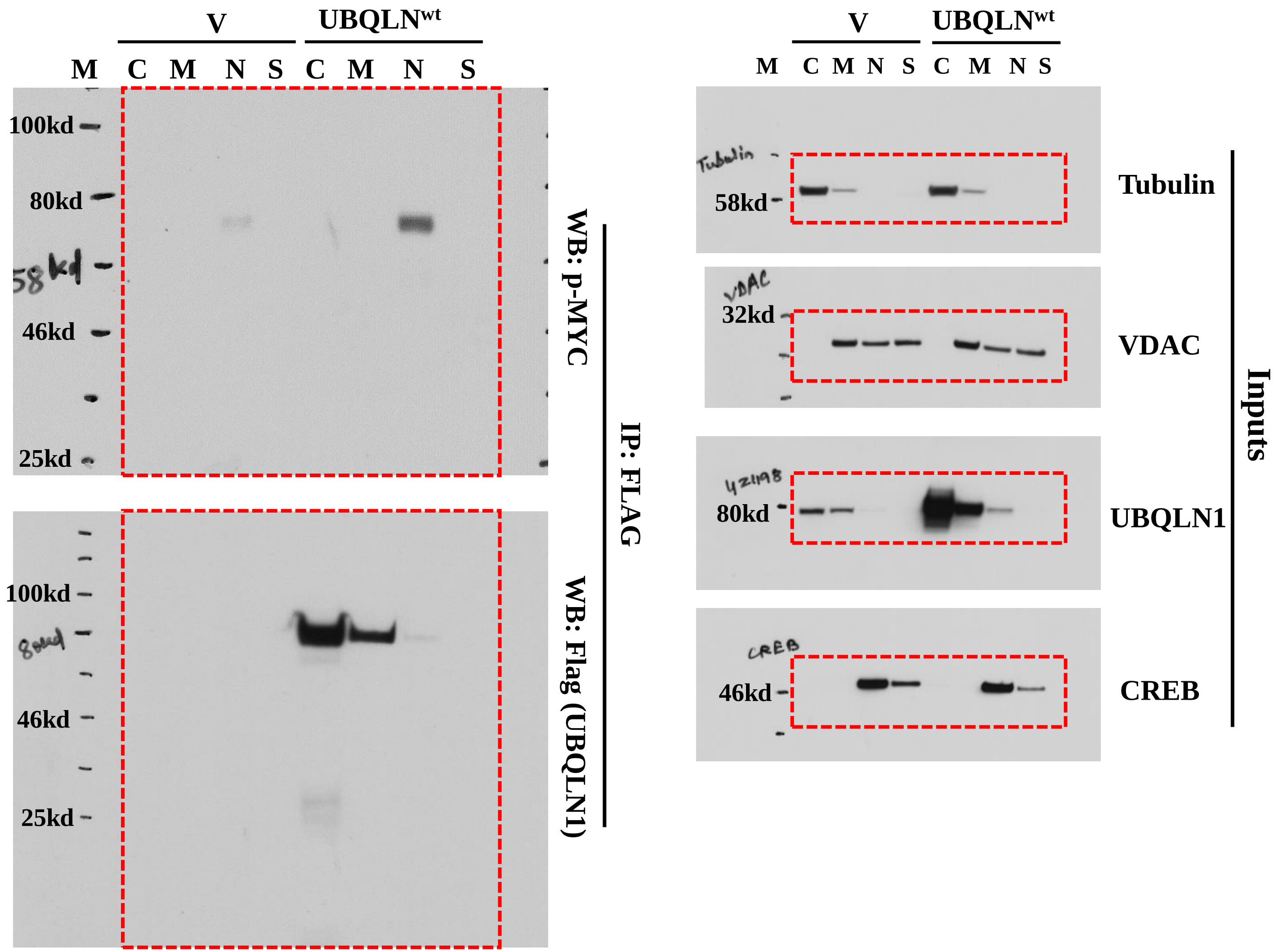

Note: M is Molecular weight marker

Figure S4D

Original Uncropped blots

Manuscript Figure

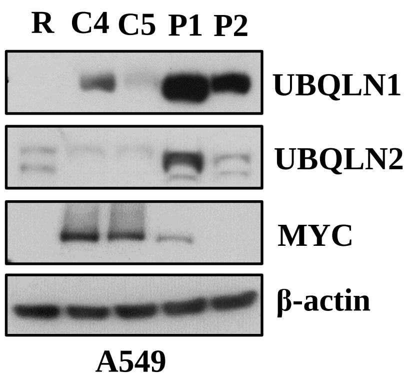

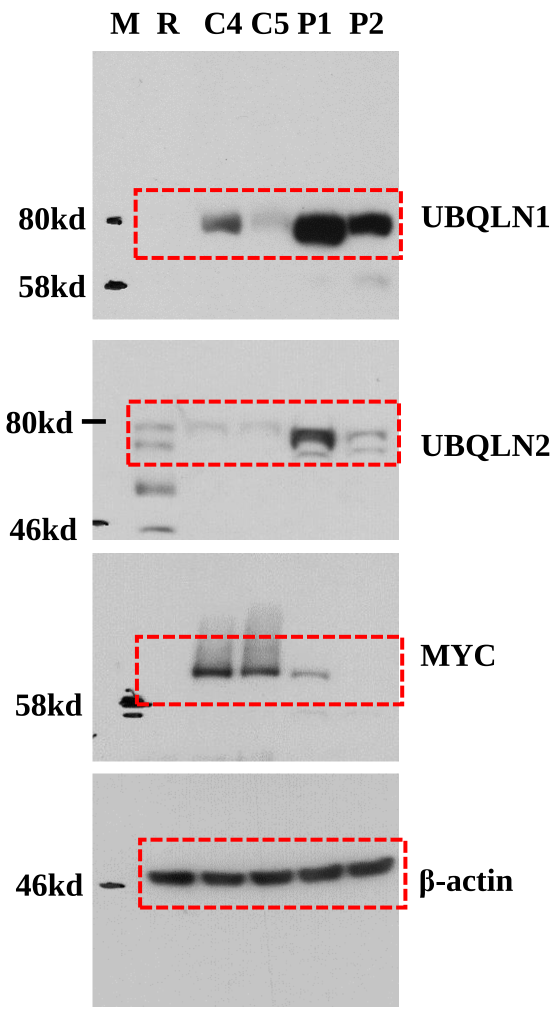

Note: M is Molecular weight marker

Figure S4E

Manuscript Figure

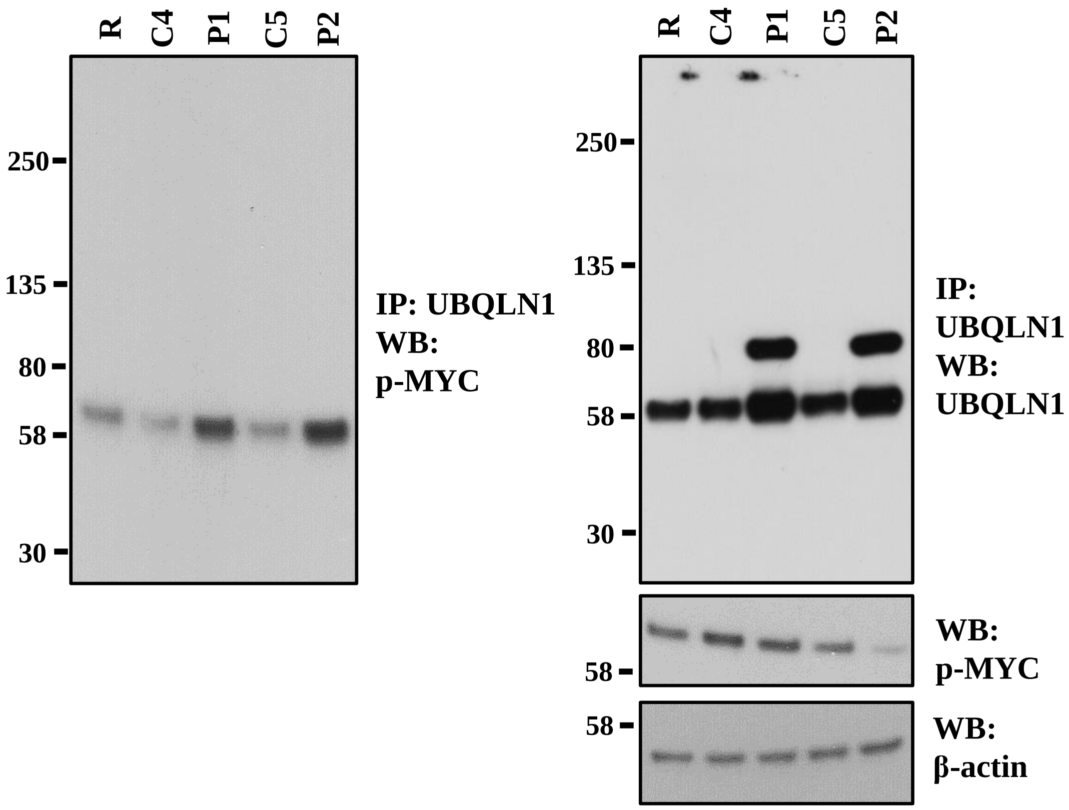

A549

Original Uncropped blots

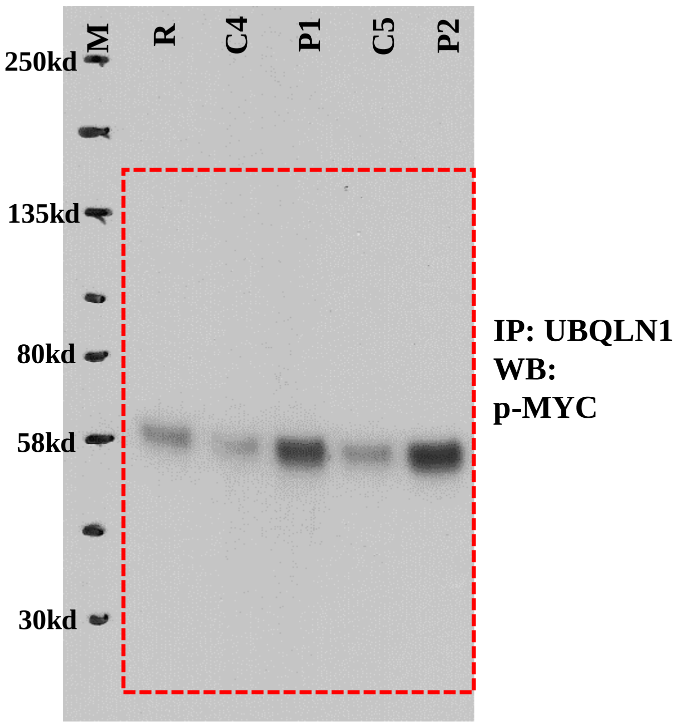

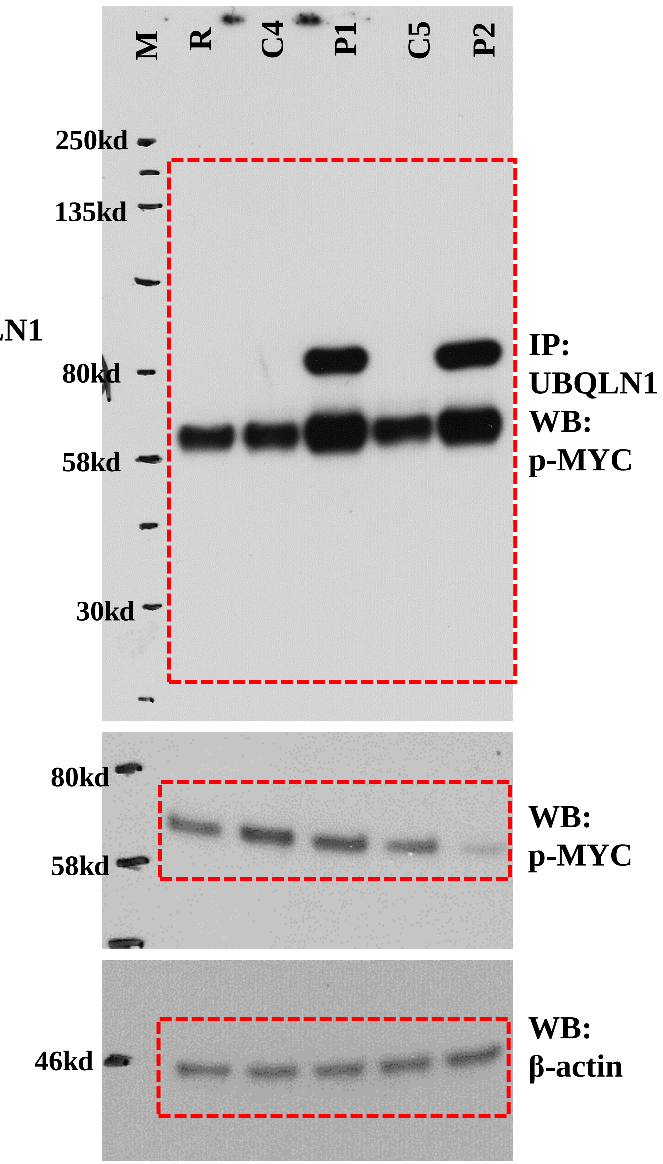

Note: M is Molecular weight marker

Figure S4F

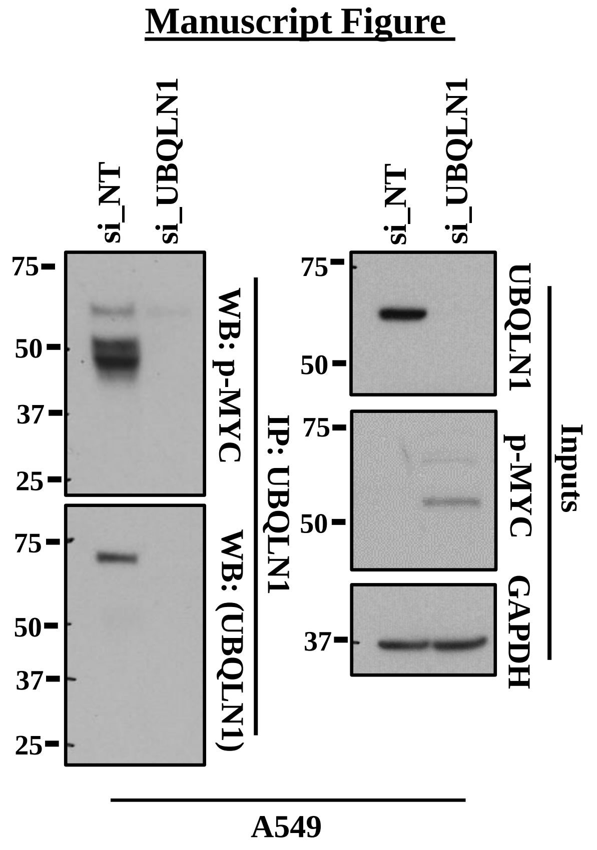

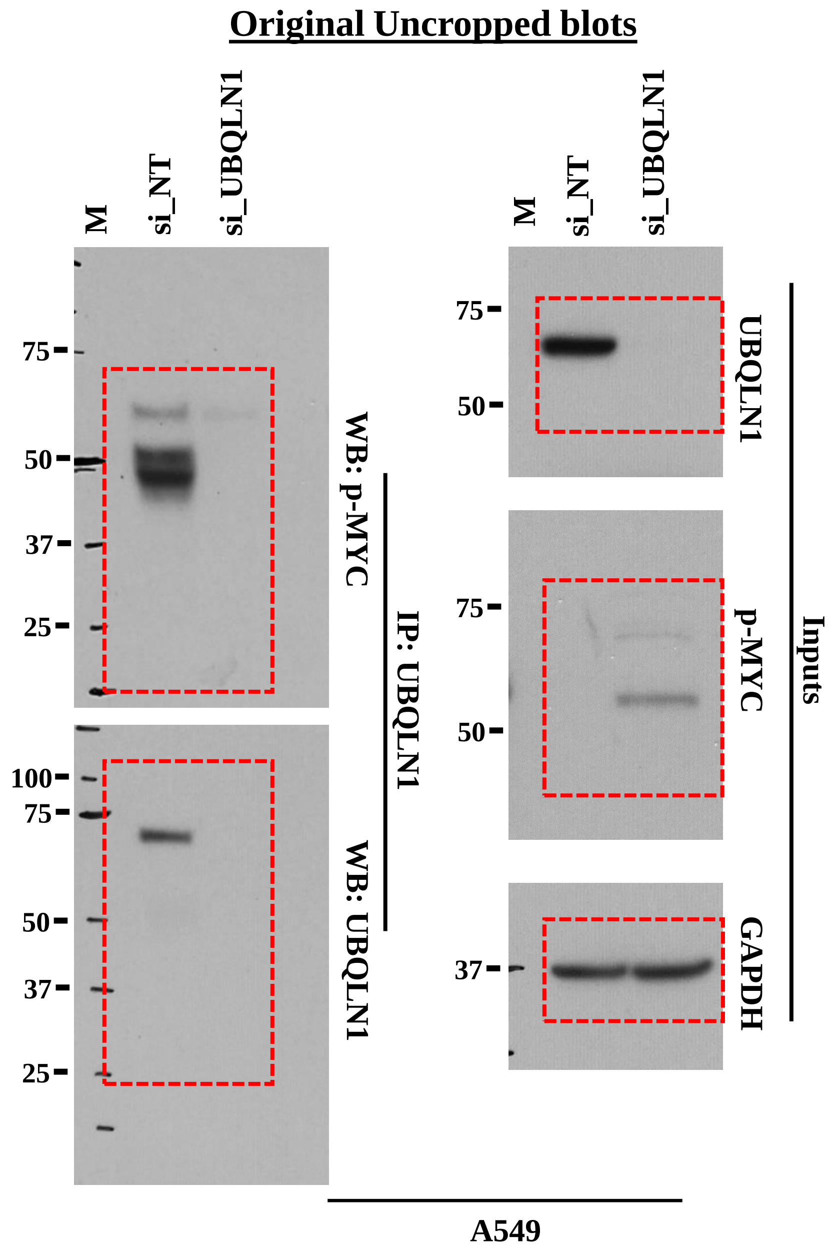

Note: M is Molecular weight marker

Figure S5

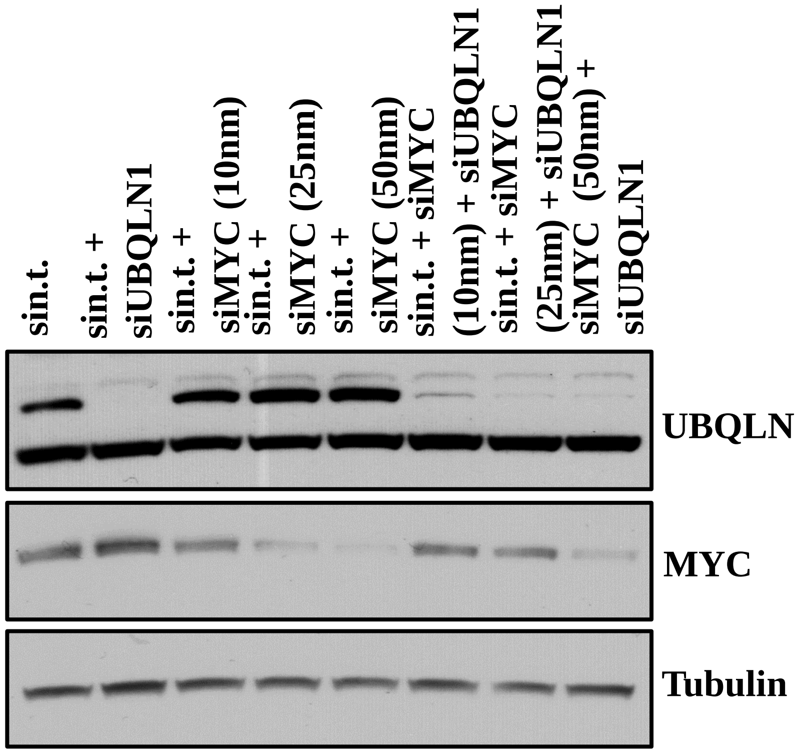

Manuscript Figure

Original Uncropped blots

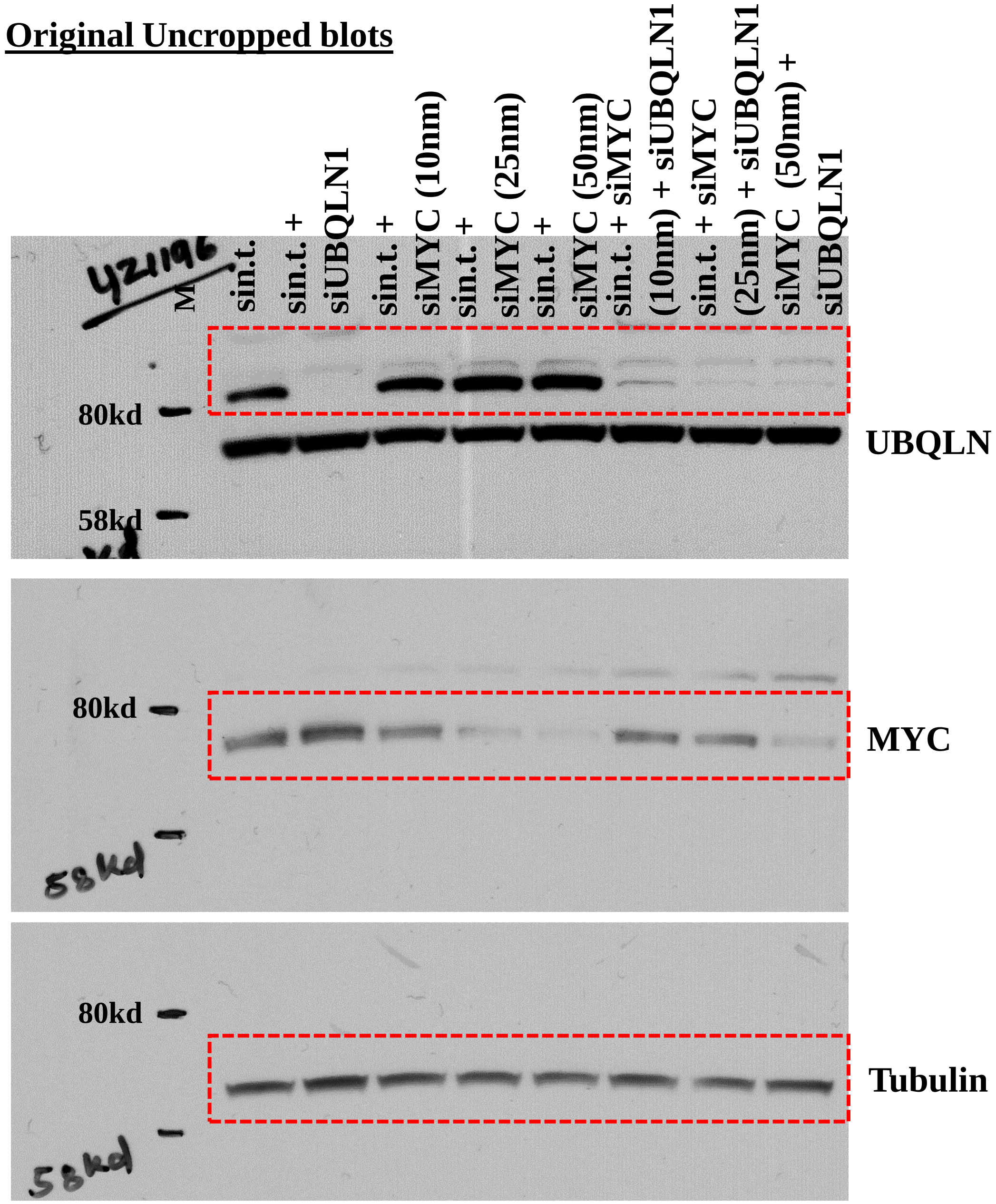

Note: M is Molecular weight marker

Figure S6A

Original Uncropped blots

Manuscript Figure

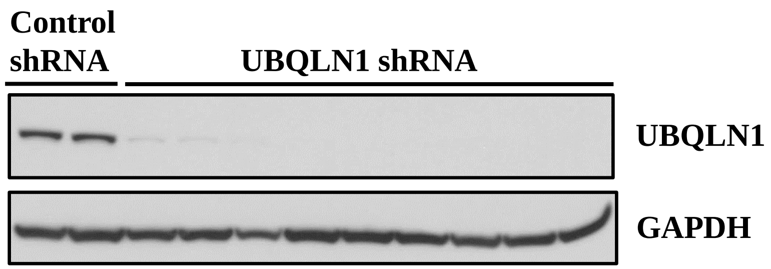

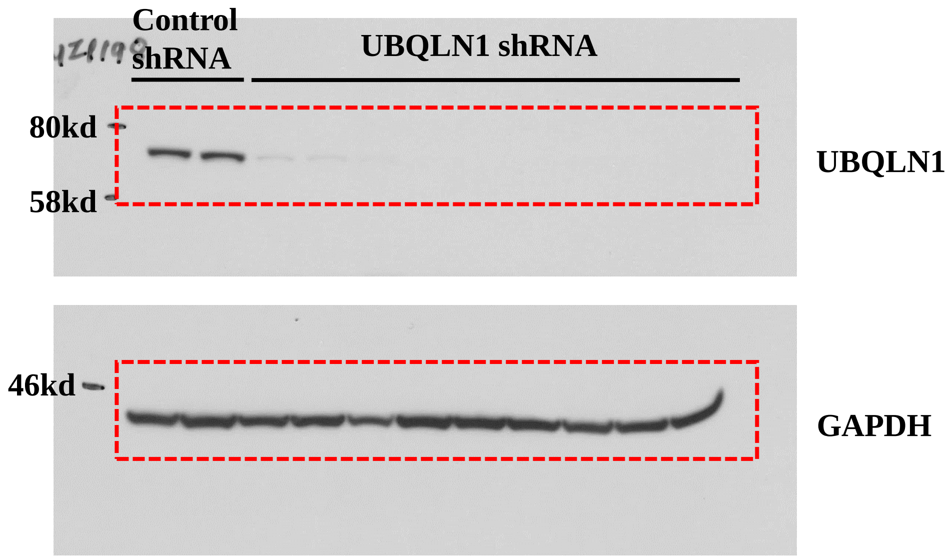

Note: M is Molecular weight marker

Figure S6B

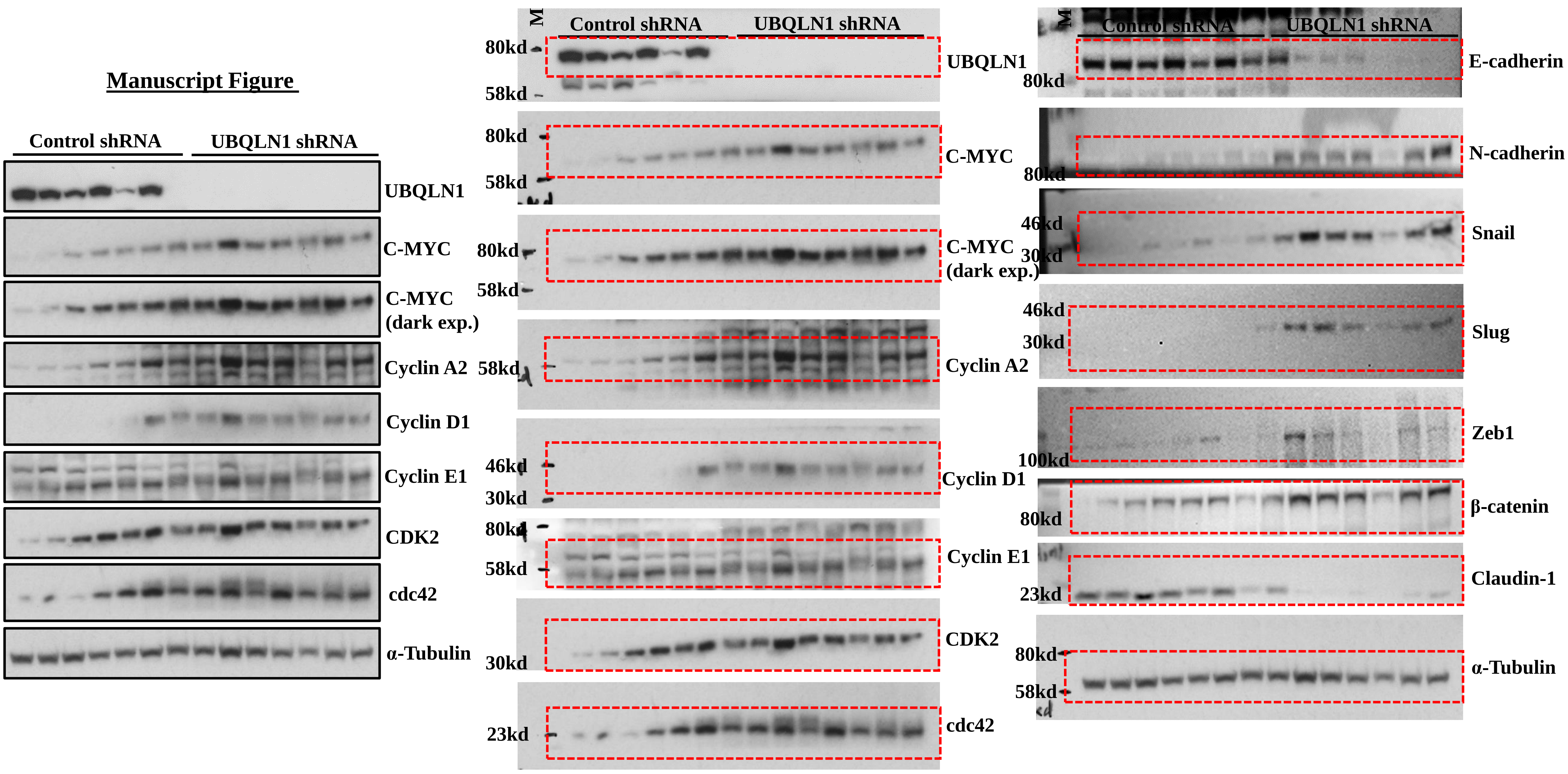

Note: M is Molecular weight marker

Supplementary Figure S7

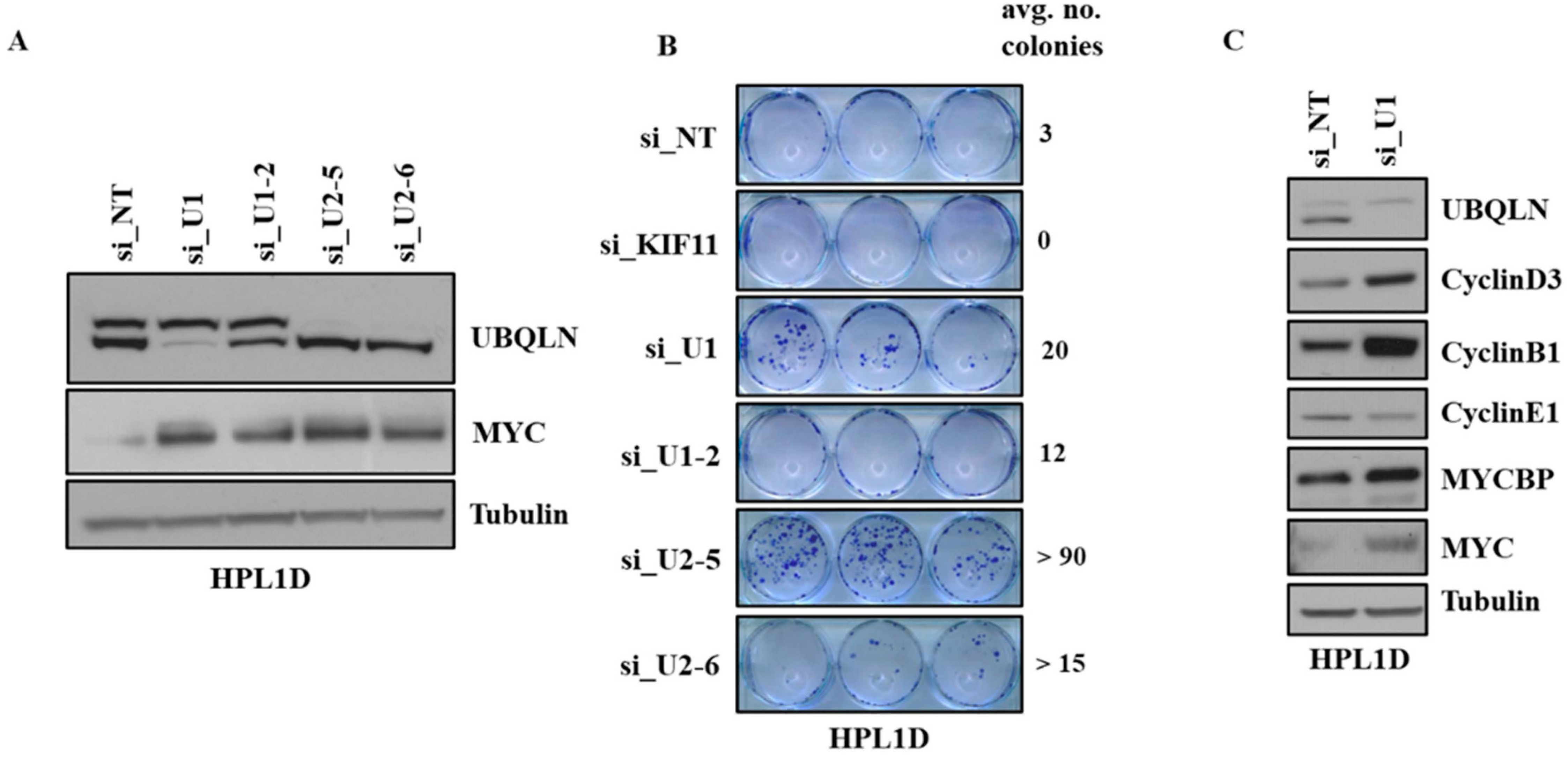

Supplementary Figure S8

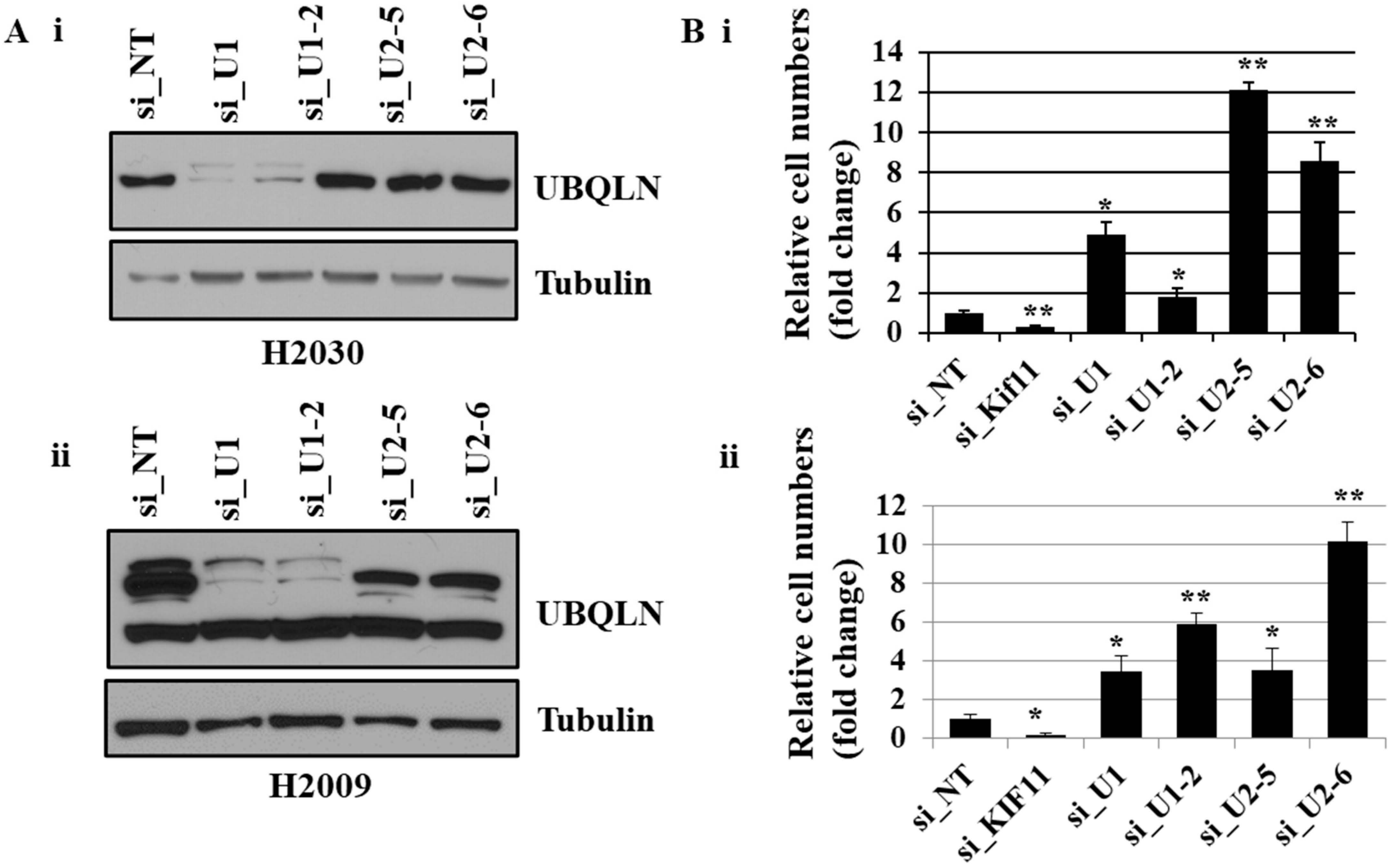

Supplement: Supplementary file 1 [file cancers-15-03389-s001.zip › cancers-2380496-supplementary.pdf]
